# Chorioamnionitis and Its Complications: A Cross-Sectional Study to Assess the Awareness of Married Women in Jeddah

**DOI:** 10.7759/cureus.29676

**Published:** 2022-09-28

**Authors:** Ghaida Abdullah Eissa, Ayman A Bukhari, Banan A Alsaif, Renad M Abualsaud, Raghad M Alhowaidi, Reham Alshehri

**Affiliations:** 1 Medicine, King Abdulaziz University Hospital, Jeddah, SAU; 2 Obstetrics and Gynaecology, King Abdulaziz University Hospital, Jeddah, SAU

**Keywords:** chorioamnionitis complications, maternal-fetal health, maternal-fetal outcomes, cesarean section, chorioamnionitis

## Abstract

Background: Chorioamnionitis (CA) is a common pregnancy complication characterized by inflammation of the placental membranes and chorion. To our knowledge, there are limited studies evaluating the awareness of CA and its complications among women in Jeddah, Saudi Arabia. This study aimed to determine the awareness of married women in Jeddah toward CA and its complications.

Materials and methods: This cross-sectional study was conducted between March 2021 and August 2021. It involved 406 women who were or have been married in Jeddah, Saudi Arabia. Data were obtained via an online survey and analyzed using IBM SPSS Statistics version 24 (IBM Corp., Armonk, NY). Different statistical tests were used for data analysis, including percentages, mean, frequency, and chi-square. Content validity and reliability were checked. Based on a woman's knowledge score, the score was classified into three levels: good knowledge level (score: 9-12), fair knowledge level (score: 5-8), and poor knowledge level (score: 0-4).

Results: Of the total number of women who participated in the study, most of them had a poor knowledge score about CA complications (49.95%), and only 8.1% had good knowledge. Among the women, 25% had previously heard about CA, while only 2.5% were diagnosed with CA, and 50% of these women delivered by cesarean section. Analysis showed a significant relationship between women who had CA and their birth method (p = 0.000). However, there was a nonsignificant difference between the females' knowledge and their age (p = 0.297), or their level of education (p = 0.099).

Conclusion: The study concluded that there was a poor level of knowledge regarding CA and its complications among women who experienced pregnancy.

## Introduction

Chorioamnionitis (CA) is a frequent pregnancy complication that refers to inflammation of the membranes and chorion of the placenta. It is also known as intra-amniotic infection and is defined as the inflammation of the placental membranes and chorion caused by ascending polymicrobial bacterial infection in the presence of membrane rupture. Although it can also happen with an intact membrane. However, there is a lot of overlap between clinical and histologic CA; the latter is a more prevalent diagnosis based on pathological abnormalities on microscopic inspection of the placenta and includes both clinical and subclinical CA [1]. It is estimated that about 2-4% of full-term pregnancies [2] and 40-70% of women who deliver preterm have CA [3,4]. A retrospective cohort study was published in 2017 in Washington to see if CA influences the incidence of respiratory distress syndrome (RDS). It reported a significant connection between CA and both composite outcomes (RDS and perinatal death) [5]. In addition, a retrospective cohort study was done in 2009 at the Moffitt-Long Hospital, San Francisco, which stated that clinical CA affects 13-60% of pregnant women with preterm membrane rupture and 1% of women who are at term [6]. Moreover, a recent study in the United States in 2019 found that a clinical diagnosis of CA was linked with an increased risk of unfavorable maternal outcomes following cesarean delivery [7]. Moreover, a prospective observational study conducted in India in 2015 concluded that in 70 out of 100 women, clinical, microbiological, histological, or a combination of these features of CA was detected [8]. A study published in Korea in 2013 found that one of the risk factors for the occurrence of adverse neonatal outcomes is CA [9]. Unfortunately, in Saudi Arabia, there has been no study on the occurrence and complications of CA. A study published in Riyadh, Saudi Arabia in 2020 demonstrated that of the 696 patients included in the study, 255 had histological data, and histological evidence for CA was found in 135 of them (52.9%) [10]. CA can pose significant neonatal risks. These include neonatal sepsis, perinatal death, and intraventricular hemorrhage [11]. As for maternal outcomes, CA increases the chances of cesarean birth by two to three times, and wound infection, bacteremia, and postpartum hemorrhage by two to four times [2,12-16].

The present study aimed to assess the knowledge level of married women who gave birth or were pregnant and suspected of CA about CA and its materno-neonatal complications, at the King Abdulaziz University Hospital (KAUH).

We hypothesized that the scarcity of data on CA and its materno-neonatal complications has led to the low awareness level of Saudi women on this condition.

## Materials and methods

Our study aimed to assess the level of knowledge about CA and its neonatal and maternal complications among females in Jeddah, Saudi Arabia, in 2021. This study adopted a quantitative descriptive cross-sectional design. It was carried out at King Abdulaziz University Hospital, Jeddah, in the western region of the Kingdom of Saudi Arabia (KSA), over a period of four months (March 2021 to August 2021). The sample size calculated for this study was 406 participants who filled out the questionnaire. Married females who gave birth or were pregnant and suspected of CA were included in the study. The sample size was calculated using a confidence interval of 95% and a margin of error of 5%. The calculations were made using the Raosoft sample size calculator (Raosoft Inc, Seattle, WA) [17]. Ethical approval was obtained from the Unit of Biomedical Research Ethics Committee, KAUH, College of Medicine (625-20).

Study population and data collection

Data were collected via an electronic Google Forms questionnaire filled by the participants of this study. The questionnaire consisted of three sections. The questionnaire started with separate questions, which included consent details and two other questions, one was about in which city the participants live, and the other one was asking if they were married or not. Thus, the respondents' socio-demographic information, which included their age, education level, income, and occupation, was covered in section one, which also covered the respondents' obstetric history, gravidity, parity, and abortion. The last question in this section was if they had any chronic diseases. By asking them numerous questions about the condition, if they had it before, and if so, what the effects were on the mother and the fetus, we evaluated their knowledge and attitudes on CA in section two. We have highlighted the most common complications of CA and some rare issues that can occur for mother and child in the last section, which was made up of yes or no questions to assess the women's knowledge about CA and its consequences.

Statistical analysis

Content validity was checked by the researcher, and the data obtained were entered into Microsoft Excel, 16th edition (Microsoft Corporation, Redmond, WA), and analyzed via IBM SPSS Statistics version 24 (IBM Corp., Armonk, NY). Qualitative data, including mode of delivery, and maternal and fetal complications, were expressed as frequencies and percentages. Numerical data like age, gravidity, parity, and abortions were expressed through measures of central tendency, including means ± standard deviations, modes, ranges, and dispersion. Furthermore, categorical data were compared via the chi-squared test to determine the relationship between CA duration and their complications, with a p-value < 0.05 to indicate a significant relationship between the variables.

## Results

The current study assessed the awareness of women who were currently pregnant or have given birth in Jeddah, toward complications of CA and its effect on the mother and the fetus. We received 406 responses and after excluding those who did not fit the eligibility criteria, a total of 322 women were included in the study. Their demographic information is shown in Table 1. Their ages ranged from 21 to 68 years old, the most frequent age group being 40 to 49 years (141, 43.8%). Furthermore, the mean age of the study sample was 44.35 ± 9.5. Most of them (302, 93,8%) were Saudi. Most of the participants (207, 64.3%) were university graduates and none were uneducated. A total of 109 (33.9%) women were housewives, and 280 (87.0%) participants had an income of more than 5000 Saudi riyals a month.

**Table 1:** Socio-demographic data

	N (%)
Age (years)	
20–29	26 (8.1)
30–39	60 (18.6)
40–49	141 (43.8)
50–59	76 (23.6)
60–69	19 (5.9)
Nationality	
Non-Saudi	20 (6.2)
Saudi	302 (93.8)
Education	
Middle school	5 (1.6)
High school	57 (17.7)
University	260 (80.7)
Occupation	
Housewife	109 (33.9)
Student	6 (1.9)
Employee	207 (64.3)
Income	
Less than 3000 Saudi riyal (SAR)	11 (3.4)
3000–5000	31 (9.6)
More than 5000 SAR	280 (87.0)
Chronic diseases	
Diabetes mellitus and hypertension	44 (13.7)
Hypothyroidism	8 (2.5)
Asthma	7 (2.2)
Others	19 (5.9)
None	244 (75.8)

Diabetes mellitus and high blood pressure were the most frequent chronic illnesses among the individuals (13.7%). Overall, 244 (75.8%) participants had three or more children, and 193 (59.9%) had a previous history of abortion. Also, when the respondents were asked about CA, 239 (75%) of them had never heard about it previously. Among participants who knew about CA, we found that social media was the most common source of information, followed by relatives and friends (36.6 and 18.75%, respectively). Only eight (2.5%) participants had a history of CA.

The maternal complications reported by participants who had suffered from the disease earlier were bleeding (50%), emergent cesarean section (50%), eclampsia (25%), and clots in different places (25%). None of the participants reported gestational diabetes mellitus or endometriosis as a complication. Regarding fetal complications, it was reported that 87.5%, 50%, 37.5%, and 12.5% suffered from premature birth, death, low birth weight, and retinopathy, respectively. Meningitis was not reported in any of the infants.

Finally, we evaluated the participating members' knowledge about complications of CA, and the results obtained are shown in Table 2. Of the participants, 158 women (49.95%) had poor knowledge, 138 women (42.9%) had fair knowledge, and only 26 women (8.1%) had good knowledge.

**Table 2:** Awareness level of chorioamnionitis

	Poor knowledge	Fair knowledge	Good knowledge
N (%)	158 (49.1)	138 (42.9)	26 (8.1)

Regarding the knowledge of the participants about CA, 224 (69.6%) of them knew that diagnosis of CA requires extensive review with a specialist. A total of 207 (64.3%) were aware that early treatment of urinary and vaginal infections helps reduce the incidence of disease. Whereas 230 (71.4%) and 210 (65.2%) of them had no idea if the mother's infection with CA could be transmitted to the fetus or not and if the disease is common in Saudi Arabia or not, respectively.

A statistical test was performed to study the relationship between awareness levels about CA and several factors. There was no statistically significant relationship between the awareness level and age (p = 0.150), education (p = 0.099), occupation (p = 0.648), income (0.714), parity (p = 0.720), and abortion (p = 324).

**Figure 1:**
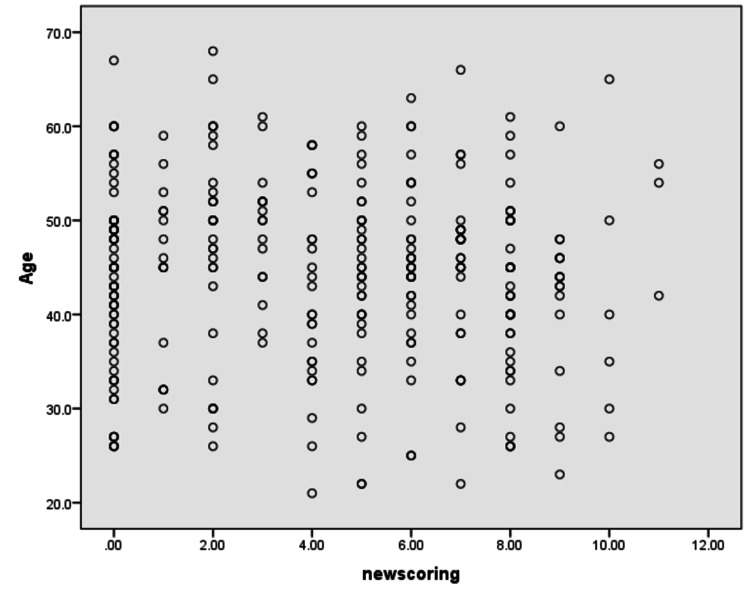
Age and knowledge score correlation

On the other hand, a significant relationship was found between those who had suffered from CA earlier and the birth method (p = 0.000). Figure 1 showed that there was a weak correlation between participants' age and knowledge score (r = -0.58; p-value = 0.297).

## Discussion

This study aimed to assess the awareness of women toward complications of CA on the mother and the fetus. Regarding the results of knowledge of the participants about complications of CA, most (49.1%) had poor knowledge, and only 8.1% had good knowledge. Our results were similar to a study reported from South Africa, by du Plessis et al., in which the majority of participants lacked knowledge regarding the screening and management of women with CA, resulting in incorrect practices in this regard. Although the result about knowledge is similar, the sample in this study and their study were completely different [18]. In this study, most of the women had university education but had poor knowledge of complications of CA. Hypothetically, this is not surprising because women with a university education participated in the present study in large numbers and filled out the questionnaire, and therefore their percentage is the largest. In fact, the questionnaire was assigned to the city of Jeddah, which is an urban city with a high level of education, and this may be the reason. This result could be attributed to the fact that some of the complications mentioned in the questionnaire were rare and hence, less likely to know about. Moreover, this study found that one of the most common complications that affect the fetus was premature birth (87.5%) followed by death (50%). This finding is in line with a case study done in Taiwan by Huang et al., who recommended that the obstetrician should be aware of Wilson-Mikity syndrome as a rare CA consequence, and this entity should raise concerns about decision-making and counseling for patients with suspicious CA, such as the requirement for intended premature birth [19]. On the other hand, many studies revealed that the gestational age at delivery is inversely connected with the CA-related morbidity and death of newborns. Morbidity is low in term babies born to women with CA [6,20,21].

Regarding the maternal complications reported by participants, bleeding (50%) and emergency cesarean section (50%) were the most commonly reported maternal complications among the participants who previously had CA. These results were consistent with some results, which showed that a higher risk of problems is linked to operations carried out in an infected surgical area (for example, a cesarean delivery of a patient with clinical CA). Similarly, a study done in Italy by Martinelli et al. found that CA is tightly correlated with postpartum hemorrhage, cesarean sections (two- to three-fold greater risk), and irregular labor progression [11,22]. Conversely, a study done in the USA suggested that a cesarean section should be reserved for standard indications and should not be performed if CA was the only indication [23].

This study had some limitations. Firstly, it depends exclusively on women living in Jeddah city, thus excluding other women in the country. Secondly, of all the participants, 141 (43.8%) were of the age group between 40 and 49 years, with a mean score of 4.35 ± 9.5 for age, which means that nearly half of the responders were from the same age group and all the other age groups composed the other half. Finally, this study was conducted using an online survey, and therefore, recall bias may be found in the responses of the participants.

## Conclusions

This study concluded that most women had poor knowledge about whether the disease is common in Saudi Arabia or not. Most of them were unaware of the possible complications in general, particularly the possibility of passing on the mother's CA infection to the fetus. However, most of the participants were well aware that the diagnosis of CA requires extensive review with a specialist, and early treatment of urinary and vaginal infections helps reduce the incidence of the disease. Furthermore, interviewing the participants in antenatal clinics would help future studies to get better results. Healthcare workers, including physicians and nurses, should provide the necessary knowledge about the possibility of having CA and its complications to pregnant women visiting antenatal clinics and provide them with other information sources such as leaflets and videos to better expand their knowledge. In addition, the Ministry of Health should provide more information on women’s health through awareness campaigns.
